# Supplementary material for: Multivariate Relationships among Carcass Traits and Proximate Composition, Lipid Profile, and Mineral Content of Longissimus lumborum of Grass-Fed Male Cattle Produced under Tropical Conditions
Source: Foods. 2021 Jun 12;10(6):1364. doi: 10.3390/foods10061364 (PMC8231157; doi:10.3390/foods10061364)
Supplement: Supplementary file 1 [file foods-10-01364-s001.zip › foods-1231610-supplementary.pdf]

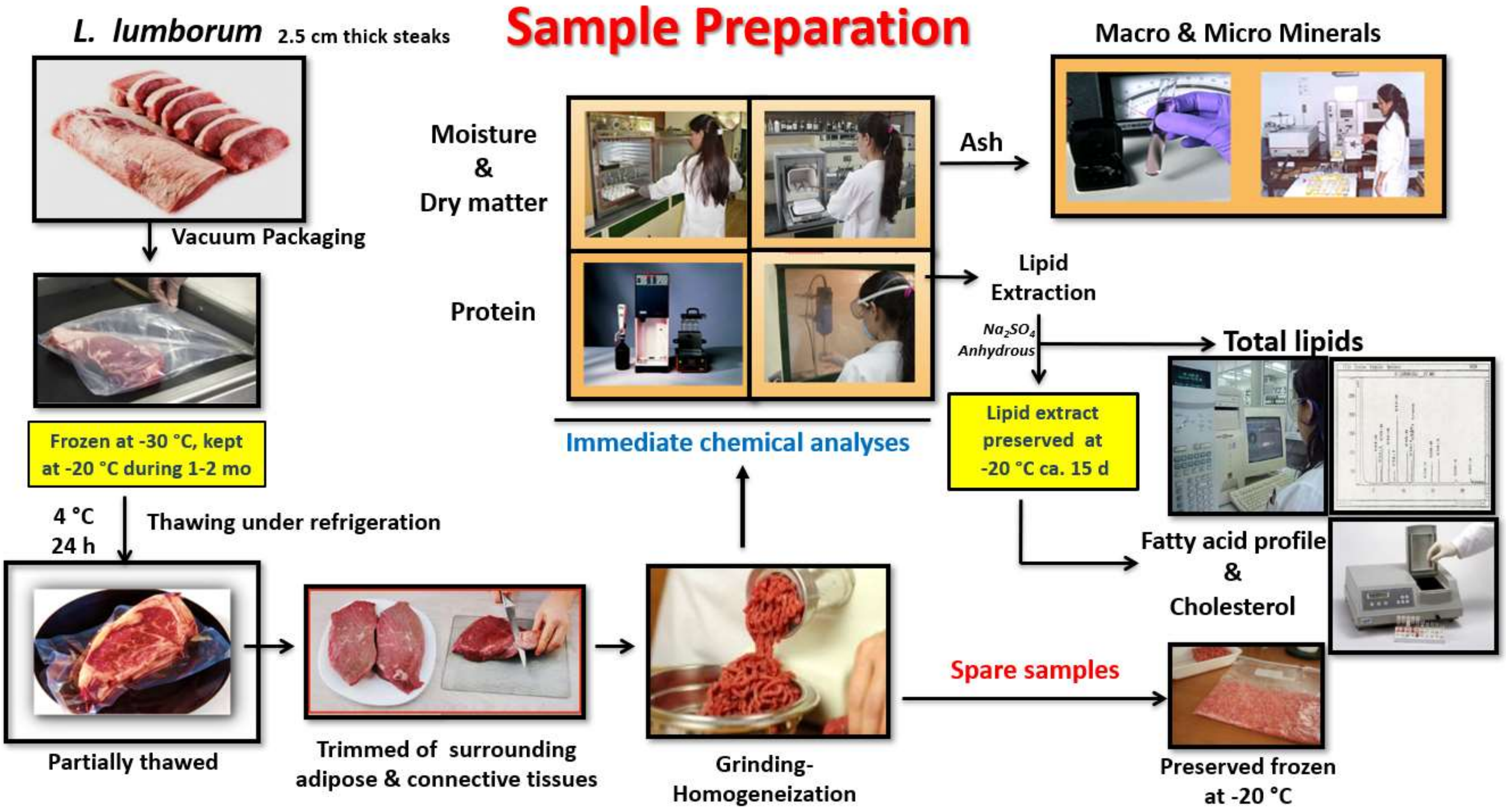

Figure S1. Graphical description of sample preparation and chemical analyses

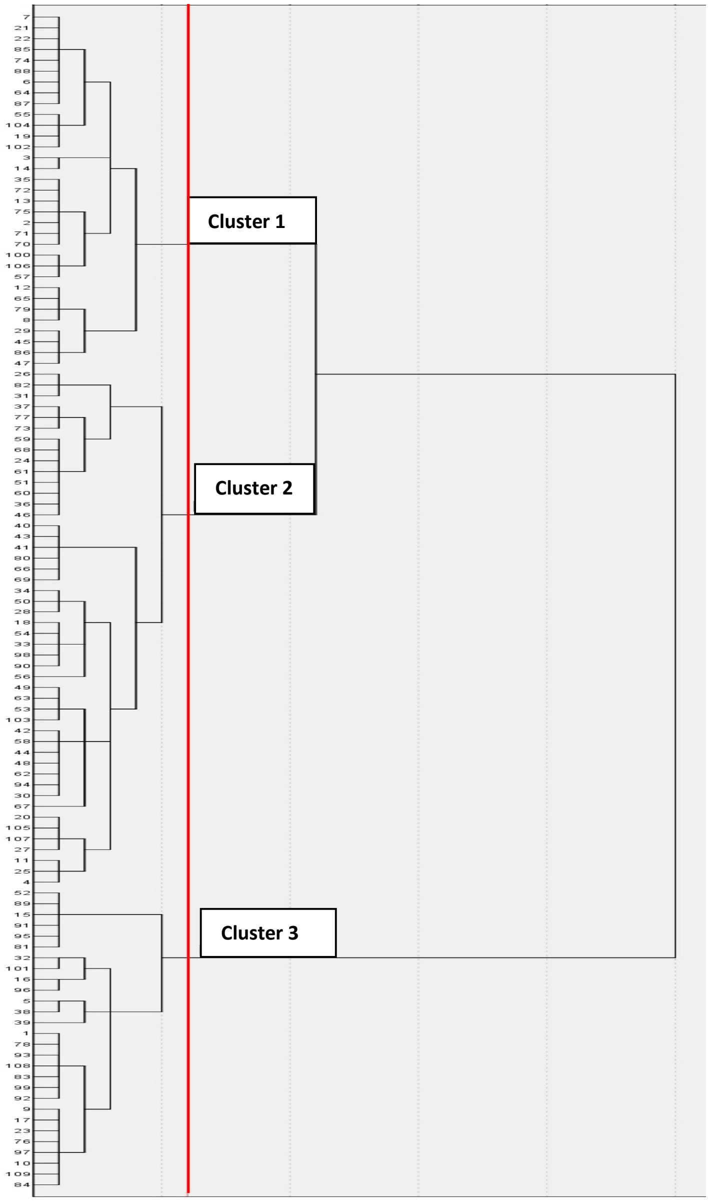

Figure S2. Dendrogram for samples from the hierarchical cluster analysis identifying clusters. The samples were numbered randomly from 1 to 109.

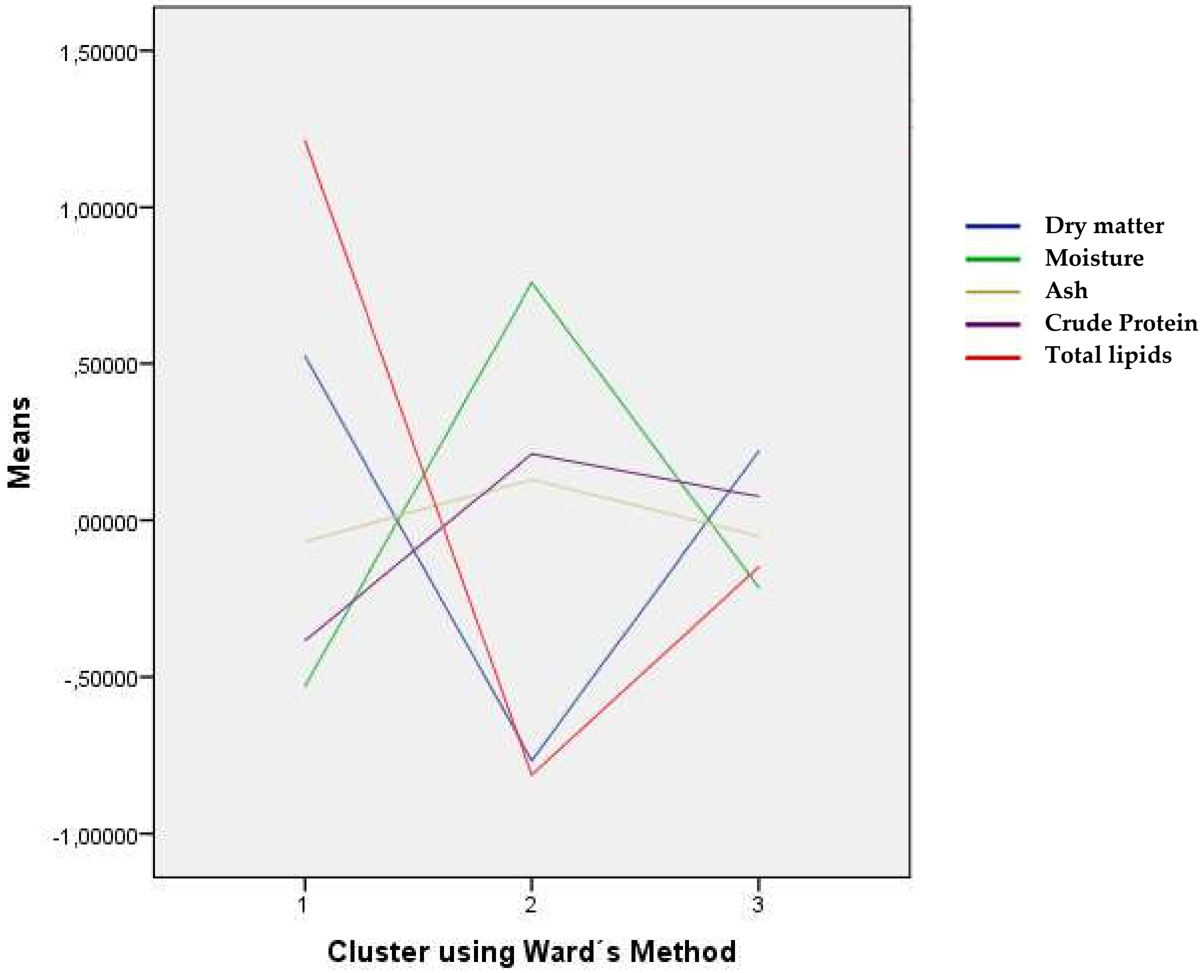

Figure S3. Projection of variables from the subgroup proximate composition by clusters

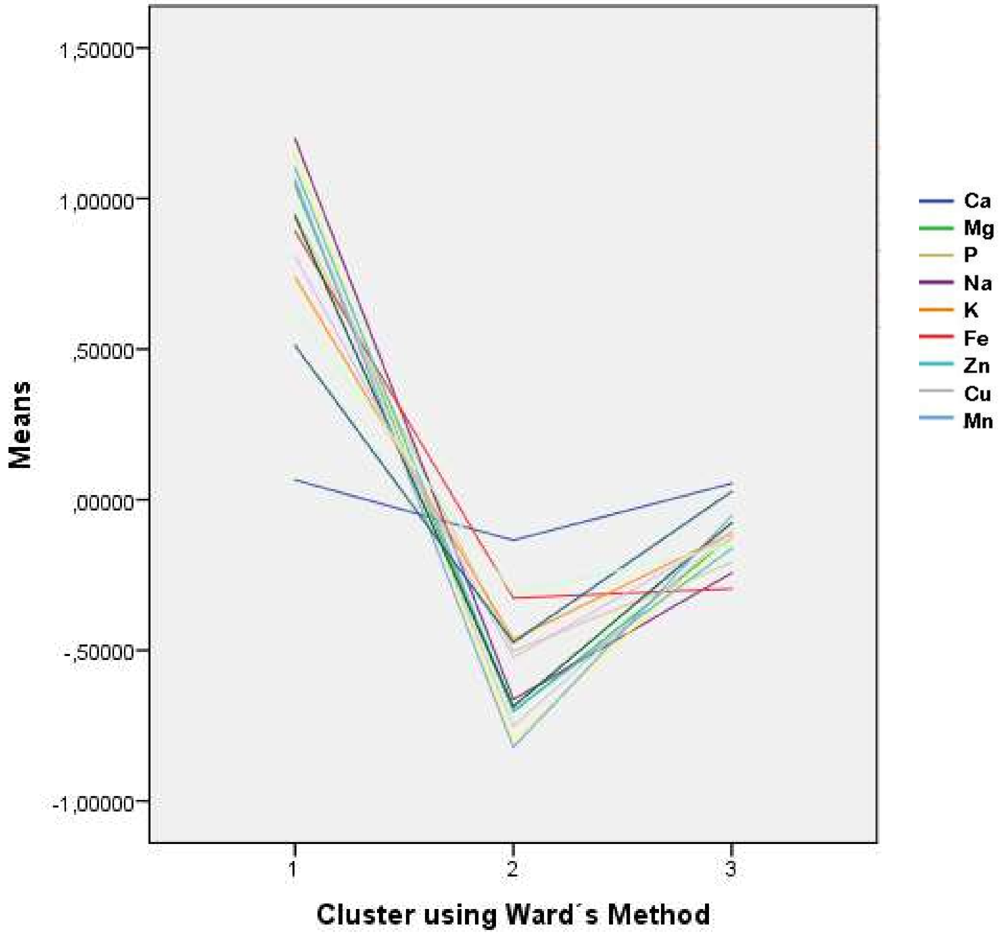

Figure S4. Projection of variables from the subgroup mineral content by clusters

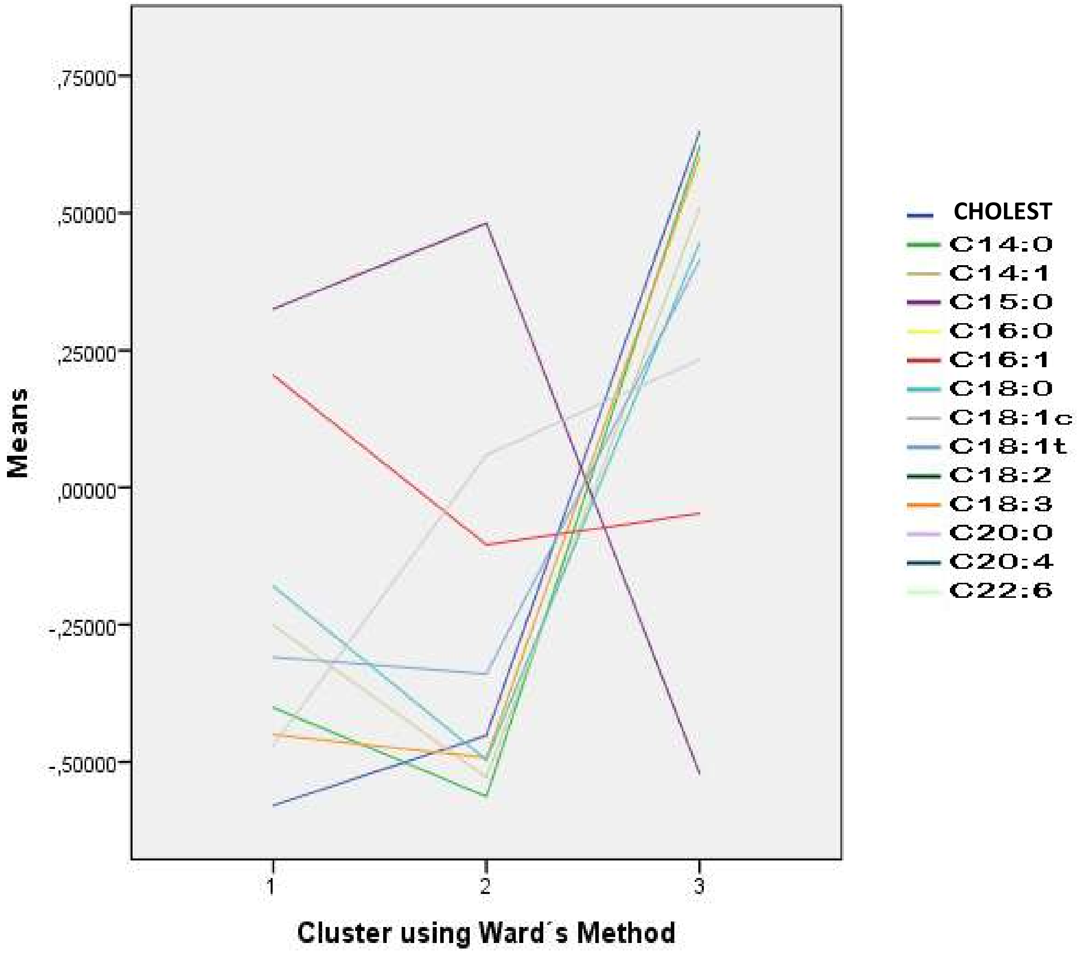

Figure S5. Projection of variables from the subgroup lipid profile by clusters

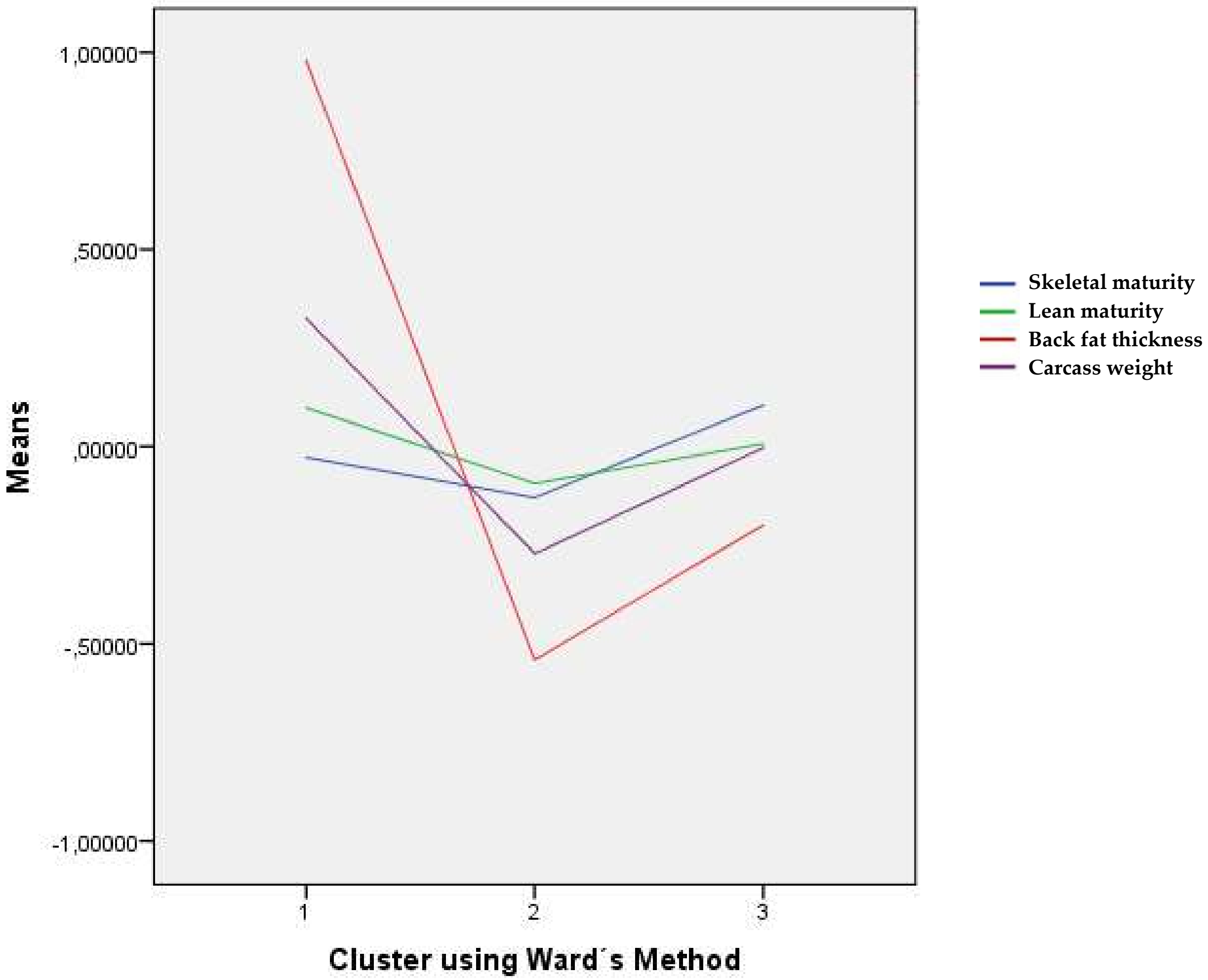

Figure S6. Projection of variables from the subgroup carcass traits by clusters
